# Supplementary material for: Unified mRNA Subcellular Localization Predictor based on machine learning techniques
Source: BMC Genomics. 2024 Feb 7;25:151. doi: 10.1186/s12864-024-10077-9 (PMC10848524; doi:10.1186/s12864-024-10077-9)
Supplement: Supplementary file 1 — Additional file 1. List of selected features for UMSLP. [file 12864_2024_10077_MOESM1_ESM.docx]

**Additional File 01:** List of selected features for UMSLP

| **Feature Name** | **Type** |
| --- | --- |
| 'AA' | Kmer |
| 'AC' | Kmer |
| 'AG' | Kmer |
| 'AT' | Kmer |
| 'CA' | Kmer |
| 'CC' | Kmer |
| 'CG' | Kmer |
| 'CT' | Kmer |
| 'GA' | Kmer |
| 'GC' | Kmer |
| 'GG' | Kmer |
| 'GT' | Kmer |
| 'TA' | Kmer |
| 'TC' | Kmer |
| 'TG' | Kmer |
| 'TT' | Kmer |
| 'AAA' | Kmer |
| 'AAC' | Kmer |
| 'AAG' | Kmer |
| 'AAT' | Kmer |
| 'ACA' | Kmer |
| 'ACC' | Kmer |
| 'ACG' | Kmer |
| 'ACT' | Kmer |
| 'AGA' | Kmer |
| 'AGC' | Kmer |
| 'AGG' | Kmer |
| 'AGT' | Kmer |
| 'ATA' | Kmer |
| 'ATC' | Kmer |
| 'ATG' | Kmer |
| 'ATT' | Kmer |
| 'CAA' | Kmer |
| 'CAC' | Kmer |
| 'CAG' | Kmer |
| 'CAT' | Kmer |
| 'CCA' | Kmer |
| 'CCC' | Kmer |
| 'CCG' | Kmer |
| 'CCT' | Kmer |
| 'CGA' | Kmer |
| 'CGC' | Kmer |
| 'CGG' | Kmer |
| 'CGT' | Kmer |
| 'CTA' | Kmer |
| 'CTC' | Kmer |
| 'CTG' | Kmer |
| 'CTT' | Kmer |
| 'GAA' | Kmer |
| 'GAC' | Kmer |
| 'GAG' | Kmer |
| 'GAT' | Kmer |
| 'GCA' | Kmer |
| 'GCC' | Kmer |
| 'GCG' | Kmer |
| 'GCT' | Kmer |
| 'GGA' | Kmer |
| 'GGC' | Kmer |
| 'GGG' | Kmer |
| 'GGT' | Kmer |
| 'GTA' | Kmer |
| 'GTC' | Kmer |
| 'GTG' | Kmer |
| 'GTT' | Kmer |
| 'TAA' | Kmer |
| 'TAC' | Kmer |
| 'TAG' | Kmer |
| 'TAT' | Kmer |
| 'TCA' | Kmer |
| 'TCC' | Kmer |
| 'TCG' | Kmer |
| 'TCT' | Kmer |
| 'TGA' | Kmer |
| 'TGC' | Kmer |
| 'TGG' | Kmer |
| 'TGT' | Kmer |
| 'TTA' | Kmer |
| 'TTC' | Kmer |
| 'TTG' | Kmer |
| 'TTT' | Kmer |
| 'AAAA' | Kmer |
| 'AAAC' | Kmer |
| 'AAAG' | Kmer |
| 'AAAT' | Kmer |
| 'AACA' | Kmer |
| 'AACC' | Kmer |
| 'AACG' | Kmer |
| 'AACT' | Kmer |
| 'AAGA' | Kmer |
| 'AAGC' | Kmer |
| 'AAGG' | Kmer |
| 'AAGT' | Kmer |
| 'AATA' | Kmer |
| 'AATC' | Kmer |
| 'AATG' | Kmer |
| 'AATT' | Kmer |
| 'ACAA' | Kmer |
| 'ACAC' | Kmer |
| 'ACAG' | Kmer |
| 'ACAT' | Kmer |
| 'ACCA' | Kmer |
| 'ACCC' | Kmer |
| 'ACCG' | Kmer |
| 'ACCT' | Kmer |
| 'ACGA' | Kmer |
| 'ACGC' | Kmer |
| 'ACGG' | Kmer |
| 'ACGT' | Kmer |
| 'ACTA' | Kmer |
| 'ACTC' | Kmer |
| 'ACTG' | Kmer |
| 'ACTT' | Kmer |
| 'AGAA' | Kmer |
| 'AGAC' | Kmer |
| 'AGAG' | Kmer |
| 'AGAT' | Kmer |
| 'AGCA' | Kmer |
| 'AGCC' | Kmer |
| 'AGCG' | Kmer |
| 'AGCT' | Kmer |
| 'AGGA' | Kmer |
| 'AGGC' | Kmer |
| 'AGGG' | Kmer |
| 'AGGT' | Kmer |
| 'AGTA' | Kmer |
| 'AGTC' | Kmer |
| 'AGTG' | Kmer |
| 'AGTT' | Kmer |
| 'ATAA' | Kmer |
| 'ATAC' | Kmer |
| 'ATAG' | Kmer |
| 'ATAT' | Kmer |
| 'ATCA' | Kmer |
| 'ATCC' | Kmer |
| 'ATCG' | Kmer |
| 'ATCT' | Kmer |
| 'ATGA' | Kmer |
| 'ATGC' | Kmer |
| 'ATGG' | Kmer |
| 'ATGT' | Kmer |
| 'ATTA' | Kmer |
| 'ATTC' | Kmer |
| 'ATTG' | Kmer |
| 'ATTT' | Kmer |
| 'CAAA' | Kmer |
| 'CAAC' | Kmer |
| 'CAAG' | Kmer |
| 'CAAT' | Kmer |
| 'CACA' | Kmer |
| 'CACC' | Kmer |
| 'CACG' | Kmer |
| 'CACT' | Kmer |
| 'CAGA' | Kmer |
| 'CAGC' | Kmer |
| 'CAGG' | Kmer |
| 'CAGT' | Kmer |
| 'CATA' | Kmer |
| 'CATC' | Kmer |
| 'CATG' | Kmer |
| 'CATT' | Kmer |
| 'CCAA' | Kmer |
| 'CCAC' | Kmer |
| 'CCAG' | Kmer |
| 'CCAT' | Kmer |
| 'CCCA' | Kmer |
| 'CCCC' | Kmer |
| 'CCCG' | Kmer |
| 'CCCT' | Kmer |
| 'CCGA' | Kmer |
| 'CCGC' | Kmer |
| 'CCGG' | Kmer |
| 'CCGT' | Kmer |
| 'CCTA' | Kmer |
| 'CCTC' | Kmer |
| 'CCTG' | Kmer |
| 'CCTT' | Kmer |
| 'CGAA' | Kmer |
| 'CGAC' | Kmer |
| 'CGAG' | Kmer |
| 'CGAT' | Kmer |
| 'CGCA' | Kmer |
| 'CGCC' | Kmer |
| 'CGCG' | Kmer |
| 'CGCT' | Kmer |
| 'CGGA' | Kmer |
| 'CGGC' | Kmer |
| 'CGGG' | Kmer |
| 'CGGT' | Kmer |
| 'CGTA' | Kmer |
| 'CGTC' | Kmer |
| 'CGTG' | Kmer |
| 'CGTT' | Kmer |
| 'CTAA' | Kmer |
| 'CTAC' | Kmer |
| 'CTAG' | Kmer |
| 'CTAT' | Kmer |
| 'CTCA' | Kmer |
| 'CTCC' | Kmer |
| 'CTCG' | Kmer |
| 'CTCT' | Kmer |
| 'CTGA' | Kmer |
| 'CTGC' | Kmer |
| 'CTGG' | Kmer |
| 'CTGT' | Kmer |
| 'CTTA' | Kmer |
| 'CTTC' | Kmer |
| 'CTTG' | Kmer |
| 'CTTT' | Kmer |
| 'GAAA' | Kmer |
| 'GAAC' | Kmer |
| 'GAAG' | Kmer |
| 'GAAT' | Kmer |
| 'GACA' | Kmer |
| 'GACC' | Kmer |
| 'GACG' | Kmer |
| 'GACT' | Kmer |
| 'GAGA' | Kmer |
| 'GAGC' | Kmer |
| 'GAGG' | Kmer |
| 'GAGT' | Kmer |
| 'GATA' | Kmer |
| 'GATC' | Kmer |
| 'GATG' | Kmer |
| 'GATT' | Kmer |
| 'GCAA' | Kmer |
| 'GCAC' | Kmer |
| 'GCAG' | Kmer |
| 'GCAT' | Kmer |
| 'GCCA' | Kmer |
| 'GCCC' | Kmer |
| 'GCCG' | Kmer |
| 'GCCT' | Kmer |
| 'GCGA' | Kmer |
| 'GCGC' | Kmer |
| 'GCGG' | Kmer |
| 'GCGT' | Kmer |
| 'GCTA' | Kmer |
| 'GCTC' | Kmer |
| 'GCTG' | Kmer |
| 'GCTT' | Kmer |
| 'GGAA' | Kmer |
| 'GGAC' | Kmer |
| 'GGAG' | Kmer |
| 'GGAT' | Kmer |
| 'GGCA' | Kmer |
| 'GGCC' | Kmer |
| 'GGCG' | Kmer |
| 'GGCT' | Kmer |
| 'GGGA' | Kmer |
| 'GGGC' | Kmer |
| 'GGGG' | Kmer |
| 'GGGT' | Kmer |
| 'GGTA' | Kmer |
| 'GGTC' | Kmer |
| 'GGTG' | Kmer |
| 'GGTT' | Kmer |
| 'GTAA' | Kmer |
| 'GTAC' | Kmer |
| 'GTAG' | Kmer |
| 'GTAT' | Kmer |
| 'GTCA' | Kmer |
| 'GTCC' | Kmer |
| 'GTCG' | Kmer |
| 'GTCT' | Kmer |
| 'GTGA' | Kmer |
| 'GTGC' | Kmer |
| 'GTGG' | Kmer |
| 'GTGT' | Kmer |
| 'GTTA' | Kmer |
| 'GTTC' | Kmer |
| 'GTTG' | Kmer |
| 'GTTT' | Kmer |
| 'TAAA' | Kmer |
| 'TAAC' | Kmer |
| 'TAAG' | Kmer |
| 'TAAT' | Kmer |
| 'TACA' | Kmer |
| 'TACC' | Kmer |
| 'TACG' | Kmer |
| 'TACT' | Kmer |
| 'TAGA' | Kmer |
| 'TAGC' | Kmer |
| 'TAGG' | Kmer |
| 'TAGT' | Kmer |
| 'TATA' | Kmer |
| 'TATC' | Kmer |
| 'TATG' | Kmer |
| 'TATT' | Kmer |
| 'TCAA' | Kmer |
| 'TCAC' | Kmer |
| 'TCAG' | Kmer |
| 'TCAT' | Kmer |
| 'TCCA' | Kmer |
| 'TCCC' | Kmer |
| 'TCCG' | Kmer |
| 'TCCT' | Kmer |
| 'TCGA' | Kmer |
| 'TCGC' | Kmer |
| 'TCGG' | Kmer |
| 'TCGT' | Kmer |
| 'TCTA' | Kmer |
| 'TCTC' | Kmer |
| 'TCTG' | Kmer |
| 'TCTT' | Kmer |
| 'TGAA' | Kmer |
| 'TGAC' | Kmer |
| 'TGAG' | Kmer |
| 'TGAT' | Kmer |
| 'TGCA' | Kmer |
| 'TGCC' | Kmer |
| 'TGCG' | Kmer |
| 'TGCT' | Kmer |
| 'TGGA' | Kmer |
| 'TGGC' | Kmer |
| 'TGGG' | Kmer |
| 'TGGT' | Kmer |
| 'TGTA' | Kmer |
| 'TGTC' | Kmer |
| 'TGTG' | Kmer |
| 'TGTT' | Kmer |
| 'TTAA' | Kmer |
| 'TTAC' | Kmer |
| 'TTAG' | Kmer |
| 'TTAT' | Kmer |
| 'TTCA' | Kmer |
| 'TTCC' | Kmer |
| 'TTCG' | Kmer |
| 'TTCT' | Kmer |
| 'TTGA' | Kmer |
| 'TTGC' | Kmer |
| 'TTGG' | Kmer |
| 'TTGT' | Kmer |
| 'TTTA' | Kmer |
| 'TTTC' | Kmer |
| 'TTTG' | Kmer |
| 'TTTT' | Kmer |
| 'AAAAA' | Kmer |
| 'AAAAC' | Kmer |
| 'AAAAG' | Kmer |
| 'AAAAT' | Kmer |
| 'AAACA' | Kmer |
| 'AAACC' | Kmer |
| 'AAACG' | Kmer |
| 'AAACT' | Kmer |
| 'AAAGA' | Kmer |
| 'AAAGC' | Kmer |
| 'AAAGG' | Kmer |
| 'AAAGT' | Kmer |
| 'AAATA' | Kmer |
| 'AAATC' | Kmer |
| 'AAATG' | Kmer |
| 'AAATT' | Kmer |
| 'AACAA' | Kmer |
| 'AACAC' | Kmer |
| 'AACAG' | Kmer |
| 'AACAT' | Kmer |
| 'AACCA' | Kmer |
| 'AACCC' | Kmer |
| 'AACCG' | Kmer |
| 'AACCT' | Kmer |
| 'AACGA' | Kmer |
| 'AACGC' | Kmer |
| 'AACGG' | Kmer |
| 'AACGT' | Kmer |
| 'AACTA' | Kmer |
| 'AACTC' | Kmer |
| 'AACTG' | Kmer |
| 'AACTT' | Kmer |
| 'AAGAA' | Kmer |
| 'AAGAC' | Kmer |
| 'AAGAG' | Kmer |
| 'AAGAT' | Kmer |
| 'AAGCA' | Kmer |
| 'AAGCC' | Kmer |
| 'AAGCG' | Kmer |
| 'AAGCT' | Kmer |
| 'AAGGA' | Kmer |
| 'AAGGC' | Kmer |
| 'AAGGG' | Kmer |
| 'AAGGT' | Kmer |
| 'AAGTA' | Kmer |
| 'AAGTC' | Kmer |
| 'AAGTG' | Kmer |
| 'AAGTT' | Kmer |
| 'AATAA' | Kmer |
| 'AATAC' | Kmer |
| 'AATAG' | Kmer |
| 'AATAT' | Kmer |
| 'AATCA' | Kmer |
| 'AATCC' | Kmer |
| 'AATCG' | Kmer |
| 'AATCT' | Kmer |
| 'AATGA' | Kmer |
| 'AATGC' | Kmer |
| 'AATGG' | Kmer |
| 'AATGT' | Kmer |
| 'AATTA' | Kmer |
| 'AATTC' | Kmer |
| 'AATTG' | Kmer |
| 'AATTT' | Kmer |
| 'ACAAA' | Kmer |
| 'ACAAC' | Kmer |
| 'ACAAG' | Kmer |
| 'ACAAT' | Kmer |
| 'ACACA' | Kmer |
| 'ACACC' | Kmer |
| 'ACACG' | Kmer |
| 'ACACT' | Kmer |
| 'ACAGA' | Kmer |
| 'ACAGC' | Kmer |
| 'ACAGG' | Kmer |
| 'ACAGT' | Kmer |
| 'ACATA' | Kmer |
| 'ACATC' | Kmer |
| 'ACATG' | Kmer |
| 'ACATT' | Kmer |
| 'ACCAA' | Kmer |
| 'ACCAC' | Kmer |
| 'ACCAG' | Kmer |
| 'ACCAT' | Kmer |
| 'ACCCA' | Kmer |
| 'ACCCC' | Kmer |
| 'ACCCG' | Kmer |
| 'ACCCT' | Kmer |
| 'ACCGA' | Kmer |
| 'ACCGC' | Kmer |
| 'ACCGG' | Kmer |
| 'ACCGT' | Kmer |
| 'ACCTA' | Kmer |
| 'ACCTC' | Kmer |
| 'ACCTG' | Kmer |
| 'ACCTT' | Kmer |
| 'ACGAA' | Kmer |
| 'ACGAC' | Kmer |
| 'ACGAG' | Kmer |
| 'ACGAT' | Kmer |
| 'ACGCA' | Kmer |
| 'ACGCC' | Kmer |
| 'ACGCG' | Kmer |
| 'ACGCT' | Kmer |
| 'ACGGA' | Kmer |
| 'ACGGC' | Kmer |
| 'ACGGG' | Kmer |
| 'ACGGT' | Kmer |
| 'ACGTA' | Kmer |
| 'ACGTC' | Kmer |
| 'ACGTG' | Kmer |
| 'ACGTT' | Kmer |
| 'ACTAA' | Kmer |
| 'ACTAC' | Kmer |
| 'ACTAG' | Kmer |
| 'ACTAT' | Kmer |
| 'ACTCA' | Kmer |
| 'ACTCC' | Kmer |
| 'ACTCG' | Kmer |
| 'ACTCT' | Kmer |
| 'ACTGA' | Kmer |
| 'ACTGC' | Kmer |
| 'ACTGG' | Kmer |
| 'ACTGT' | Kmer |
| 'ACTTA' | Kmer |
| 'ACTTC' | Kmer |
| 'ACTTG' | Kmer |
| 'ACTTT' | Kmer |
| 'AGAAA' | Kmer |
| 'AGAAC' | Kmer |
| 'AGAAG' | Kmer |
| 'AGAAT' | Kmer |
| 'AGACA' | Kmer |
| 'AGACC' | Kmer |
| 'AGACG' | Kmer |
| 'AGACT' | Kmer |
| 'AGAGA' | Kmer |
| 'AGAGC' | Kmer |
| 'AGAGG' | Kmer |
| 'AGAGT' | Kmer |
| 'AGATA' | Kmer |
| 'AGATC' | Kmer |
| 'AGATG' | Kmer |
| 'AGATT' | Kmer |
| 'AGCAA' | Kmer |
| 'AGCAC' | Kmer |
| 'AGCAG' | Kmer |
| 'AGCAT' | Kmer |
| 'AGCCA' | Kmer |
| 'AGCCC' | Kmer |
| 'AGCCG' | Kmer |
| 'AGCCT' | Kmer |
| 'AGCGA' | Kmer |
| 'AGCGC' | Kmer |
| 'AGCGG' | Kmer |
| 'AGCGT' | Kmer |
| 'AGCTA' | Kmer |
| 'AGCTC' | Kmer |
| 'AGCTG' | Kmer |
| 'AGCTT' | Kmer |
| 'AGGAA' | Kmer |
| 'AGGAC' | Kmer |
| 'AGGAG' | Kmer |
| 'AGGAT' | Kmer |
| 'AGGCA' | Kmer |
| 'AGGCC' | Kmer |
| 'AGGCG' | Kmer |
| 'AGGCT' | Kmer |
| 'AGGGA' | Kmer |
| 'AGGGC' | Kmer |
| 'AGGGG' | Kmer |
| 'AGGGT' | Kmer |
| 'AGGTA' | Kmer |
| 'AGGTC' | Kmer |
| 'AGGTG' | Kmer |
| 'AGGTT' | Kmer |
| 'AGTAA' | Kmer |
| 'AGTAC' | Kmer |
| 'AGTAG' | Kmer |
| 'AGTAT' | Kmer |
| 'AGTCA' | Kmer |
| 'AGTCC' | Kmer |
| 'AGTCG' | Kmer |
| 'AGTCT' | Kmer |
| 'AGTGA' | Kmer |
| 'AGTGC' | Kmer |
| 'AGTGG' | Kmer |
| 'AGTGT' | Kmer |
| 'AGTTA' | Kmer |
| 'AGTTC' | Kmer |
| 'AGTTG' | Kmer |
| 'AGTTT' | Kmer |
| 'ATAAA' | Kmer |
| 'ATAAC' | Kmer |
| 'ATAAG' | Kmer |
| 'ATAAT' | Kmer |
| 'ATACA' | Kmer |
| 'ATACC' | Kmer |
| 'ATACG' | Kmer |
| 'ATACT' | Kmer |
| 'ATAGA' | Kmer |
| 'ATAGC' | Kmer |
| 'ATAGG' | Kmer |
| 'ATAGT' | Kmer |
| 'ATATA' | Kmer |
| 'ATATC' | Kmer |
| 'ATATG' | Kmer |
| 'ATATT' | Kmer |
| 'ATCAA' | Kmer |
| 'ATCAC' | Kmer |
| 'ATCAG' | Kmer |
| 'ATCAT' | Kmer |
| 'ATCCA' | Kmer |
| 'ATCCC' | Kmer |
| 'ATCCG' | Kmer |
| 'ATCCT' | Kmer |
| 'ATCGA' | Kmer |
| 'ATCGC' | Kmer |
| 'ATCGG' | Kmer |
| 'ATCGT' | Kmer |
| 'ATCTA' | Kmer |
| 'ATCTC' | Kmer |
| 'ATCTG' | Kmer |
| 'ATCTT' | Kmer |
| 'ATGAA' | Kmer |
| 'ATGAC' | Kmer |
| 'ATGAG' | Kmer |
| 'ATGAT' | Kmer |
| 'ATGCA' | Kmer |
| 'ATGCC' | Kmer |
| 'ATGCG' | Kmer |
| 'ATGCT' | Kmer |
| 'ATGGA' | Kmer |
| 'ATGGC' | Kmer |
| 'ATGGG' | Kmer |
| 'ATGGT' | Kmer |
| 'ATGTA' | Kmer |
| 'ATGTC' | Kmer |
| 'ATGTG' | Kmer |
| 'ATGTT' | Kmer |
| 'ATTAA' | Kmer |
| 'ATTAC' | Kmer |
| 'ATTAG' | Kmer |
| 'ATTAT' | Kmer |
| 'ATTCA' | Kmer |
| 'ATTCC' | Kmer |
| 'ATTCG' | Kmer |
| 'ATTCT' | Kmer |
| 'ATTGA' | Kmer |
| 'ATTGC' | Kmer |
| 'ATTGG' | Kmer |
| 'ATTGT' | Kmer |
| 'ATTTA' | Kmer |
| 'ATTTC' | Kmer |
| 'ATTTG' | Kmer |
| 'ATTTT' | Kmer |
| 'CAAAA' | Kmer |
| 'CAAAC' | Kmer |
| 'CAAAG' | Kmer |
| 'CAAAT' | Kmer |
| 'CAACA' | Kmer |
| 'CAACC' | Kmer |
| 'CAACG' | Kmer |
| 'CAACT' | Kmer |
| 'CAAGA' | Kmer |
| 'CAAGC' | Kmer |
| 'CAAGG' | Kmer |
| 'CAAGT' | Kmer |
| 'CAATA' | Kmer |
| 'CAATC' | Kmer |
| 'CAATG' | Kmer |
| 'CAATT' | Kmer |
| 'CACAA' | Kmer |
| 'CACAC' | Kmer |
| 'CACAG' | Kmer |
| 'CACAT' | Kmer |
| 'CACCA' | Kmer |
| 'CACCC' | Kmer |
| 'CACCG' | Kmer |
| 'CACCT' | Kmer |
| 'CACGA' | Kmer |
| 'CACGC' | Kmer |
| 'CACGG' | Kmer |
| 'CACGT' | Kmer |
| 'CACTA' | Kmer |
| 'CACTC' | Kmer |
| 'CACTG' | Kmer |
| 'CACTT' | Kmer |
| 'CAGAA' | Kmer |
| 'CAGAC' | Kmer |
| 'CAGAG' | Kmer |
| 'CAGAT' | Kmer |
| 'CAGCA' | Kmer |
| 'CAGCC' | Kmer |
| 'CAGCG' | Kmer |
| 'CAGCT' | Kmer |
| 'CAGGA' | Kmer |
| 'CAGGC' | Kmer |
| 'CAGGG' | Kmer |
| 'CAGGT' | Kmer |
| 'CAGTA' | Kmer |
| 'CAGTC' | Kmer |
| 'CAGTG' | Kmer |
| 'CAGTT' | Kmer |
| 'CATAA' | Kmer |
| 'CATAC' | Kmer |
| 'CATAG' | Kmer |
| 'CATAT' | Kmer |
| 'CATCA' | Kmer |
| 'CATCC' | Kmer |
| 'CATCG' | Kmer |
| 'CATCT' | Kmer |
| 'CATGA' | Kmer |
| 'CATGC' | Kmer |
| 'CATGG' | Kmer |
| 'CATGT' | Kmer |
| 'CATTA' | Kmer |
| 'CATTC' | Kmer |
| 'CATTG' | Kmer |
| 'CATTT' | Kmer |
| 'CCAAA' | Kmer |
| 'CCAAC' | Kmer |
| 'CCAAG' | Kmer |
| 'CCAAT' | Kmer |
| 'CCACA' | Kmer |
| 'CCACC' | Kmer |
| 'CCACG' | Kmer |
| 'CCACT' | Kmer |
| 'CCAGA' | Kmer |
| 'CCAGC' | Kmer |
| 'CCAGG' | Kmer |
| 'CCAGT' | Kmer |
| 'CCATA' | Kmer |
| 'CCATC' | Kmer |
| 'CCATG' | Kmer |
| 'CCATT' | Kmer |
| 'CCCAA' | Kmer |
| 'CCCAC' | Kmer |
| 'CCCAG' | Kmer |
| 'CCCAT' | Kmer |
| 'CCCCA' | Kmer |
| 'CCCCC' | Kmer |
| 'CCCCG' | Kmer |
| 'CCCCT' | Kmer |
| 'CCCGA' | Kmer |
| 'CCCGC' | Kmer |
| 'CCCGG' | Kmer |
| 'CCCGT' | Kmer |
| 'CCCTA' | Kmer |
| 'CCCTC' | Kmer |
| 'CCCTG' | Kmer |
| 'CCCTT' | Kmer |
| 'CCGAA' | Kmer |
| 'CCGAC' | Kmer |
| 'CCGAG' | Kmer |
| 'CCGAT' | Kmer |
| 'CCGCA' | Kmer |
| 'CCGCC' | Kmer |
| 'CCGCG' | Kmer |
| 'CCGCT' | Kmer |
| 'CCGGA' | Kmer |
| 'CCGGC' | Kmer |
| 'CCGGG' | Kmer |
| 'CCGGT' | Kmer |
| 'CCGTA' | Kmer |
| 'CCGTC' | Kmer |
| 'CCGTG' | Kmer |
| 'CCGTT' | Kmer |
| 'CCTAA' | Kmer |
| 'CCTAC' | Kmer |
| 'CCTAG' | Kmer |
| 'CCTAT' | Kmer |
| 'CCTCA' | Kmer |
| 'CCTCC' | Kmer |
| 'CCTCG' | Kmer |
| 'CCTCT' | Kmer |
| 'CCTGA' | Kmer |
| 'CCTGC' | Kmer |
| 'CCTGG' | Kmer |
| 'CCTGT' | Kmer |
| 'CCTTA' | Kmer |
| 'CCTTC' | Kmer |
| 'CCTTG' | Kmer |
| 'CCTTT' | Kmer |
| 'CGAAA' | Kmer |
| 'CGAAC' | Kmer |
| 'CGAAG' | Kmer |
| 'CGAAT' | Kmer |
| 'CGACA' | Kmer |
| 'CGACC' | Kmer |
| 'CGACG' | Kmer |
| 'CGACT' | Kmer |
| 'CGAGA' | Kmer |
| 'CGAGC' | Kmer |
| 'CGAGG' | Kmer |
| 'CGAGT' | Kmer |
| 'CGATA' | Kmer |
| 'CGATC' | Kmer |
| 'CGATG' | Kmer |
| 'CGATT' | Kmer |
| 'CGCAA' | Kmer |
| 'CGCAC' | Kmer |
| 'CGCAG' | Kmer |
| 'CGCAT' | Kmer |
| 'CGCCA' | Kmer |
| 'CGCCC' | Kmer |
| 'CGCCG' | Kmer |
| 'CGCCT' | Kmer |
| 'CGCGA' | Kmer |
| 'CGCGC' | Kmer |
| 'CGCGG' | Kmer |
| 'CGCGT' | Kmer |
| 'CGCTA' | Kmer |
| 'CGCTC' | Kmer |
| 'CGCTG' | Kmer |
| 'CGCTT' | Kmer |
| 'CGGAA' | Kmer |
| 'CGGAC' | Kmer |
| 'CGGAG' | Kmer |
| 'CGGAT' | Kmer |
| 'CGGCA' | Kmer |
| 'CGGCC' | Kmer |
| 'CGGCG' | Kmer |
| 'CGGCT' | Kmer |
| 'CGGGA' | Kmer |
| 'CGGGC' | Kmer |
| 'CGGGG' | Kmer |
| 'CGGGT' | Kmer |
| 'CGGTA' | Kmer |
| 'CGGTC' | Kmer |
| 'CGGTG' | Kmer |
| 'CGGTT' | Kmer |
| 'CGTAA' | Kmer |
| 'CGTAC' | Kmer |
| 'CGTAG' | Kmer |
| 'CGTAT' | Kmer |
| 'CGTCA' | Kmer |
| 'CGTCC' | Kmer |
| 'CGTCG' | Kmer |
| 'CGTCT' | Kmer |
| 'CGTGA' | Kmer |
| 'CGTGC' | Kmer |
| 'CGTGG' | Kmer |
| 'CGTGT' | Kmer |
| 'CGTTA' | Kmer |
| 'CGTTC' | Kmer |
| 'CGTTG' | Kmer |
| 'CGTTT' | Kmer |
| 'CTAAA' | Kmer |
| 'CTAAC' | Kmer |
| 'CTAAG' | Kmer |
| 'CTAAT' | Kmer |
| 'CTACA' | Kmer |
| 'CTACC' | Kmer |
| 'CTACG' | Kmer |
| 'CTACT' | Kmer |
| 'CTAGA' | Kmer |
| 'CTAGC' | Kmer |
| 'CTAGG' | Kmer |
| 'CTAGT' | Kmer |
| 'CTATA' | Kmer |
| 'CTATC' | Kmer |
| 'CTATG' | Kmer |
| 'CTATT' | Kmer |
| 'CTCAA' | Kmer |
| 'CTCAC' | Kmer |
| 'CTCAG' | Kmer |
| 'CTCAT' | Kmer |
| 'CTCCA' | Kmer |
| 'CTCCC' | Kmer |
| 'CTCCG' | Kmer |
| 'CTCCT' | Kmer |
| 'CTCGA' | Kmer |
| 'CTCGC' | Kmer |
| 'CTCGG' | Kmer |
| 'CTCGT' | Kmer |
| 'CTCTA' | Kmer |
| 'CTCTC' | Kmer |
| 'CTCTG' | Kmer |
| 'CTCTT' | Kmer |
| 'CTGAA' | Kmer |
| 'CTGAC' | Kmer |
| 'CTGAG' | Kmer |
| 'CTGAT' | Kmer |
| 'CTGCA' | Kmer |
| 'CTGCC' | Kmer |
| 'CTGCG' | Kmer |
| 'CTGCT' | Kmer |
| 'CTGGA' | Kmer |
| 'CTGGC' | Kmer |
| 'CTGGG' | Kmer |
| 'CTGGT' | Kmer |
| 'CTGTA' | Kmer |
| 'CTGTC' | Kmer |
| 'CTGTG' | Kmer |
| 'CTGTT' | Kmer |
| 'CTTAA' | Kmer |
| 'CTTAC' | Kmer |
| 'CTTAG' | Kmer |
| 'CTTAT' | Kmer |
| 'CTTCA' | Kmer |
| 'CTTCC' | Kmer |
| 'CTTCG' | Kmer |
| 'CTTCT' | Kmer |
| 'CTTGA' | Kmer |
| 'CTTGC' | Kmer |
| 'CTTGG' | Kmer |
| 'CTTGT' | Kmer |
| 'CTTTA' | Kmer |
| 'CTTTC' | Kmer |
| 'CTTTG' | Kmer |
| 'CTTTT' | Kmer |
| 'GAAAA' | Kmer |
| 'GAAAC' | Kmer |
| 'GAAAG' | Kmer |
| 'GAAAT' | Kmer |
| 'GAACA' | Kmer |
| 'GAACC' | Kmer |
| 'GAACG' | Kmer |
| 'GAACT' | Kmer |
| 'GAAGA' | Kmer |
| 'GAAGC' | Kmer |
| 'GAAGG' | Kmer |
| 'GAAGT' | Kmer |
| 'GAATA' | Kmer |
| 'GAATC' | Kmer |
| 'GAATG' | Kmer |
| 'GAATT' | Kmer |
| 'GACAA' | Kmer |
| 'GACAC' | Kmer |
| 'GACAG' | Kmer |
| 'GACAT' | Kmer |
| 'GACCA' | Kmer |
| 'GACCC' | Kmer |
| 'GACCG' | Kmer |
| 'GACCT' | Kmer |
| 'GACGA' | Kmer |
| 'GACGC' | Kmer |
| 'GACGG' | Kmer |
| 'GACGT' | Kmer |
| 'GACTA' | Kmer |
| 'GACTC' | Kmer |
| 'GACTG' | Kmer |
| 'GACTT' | Kmer |
| 'GAGAA' | Kmer |
| 'GAGAC' | Kmer |
| 'GAGAG' | Kmer |
| 'GAGAT' | Kmer |
| 'GAGCA' | Kmer |
| 'GAGCC' | Kmer |
| 'GAGCG' | Kmer |
| 'GAGCT' | Kmer |
| 'GAGGA' | Kmer |
| 'GAGGC' | Kmer |
| 'GAGGG' | Kmer |
| 'GAGGT' | Kmer |
| 'GAGTA' | Kmer |
| 'GAGTC' | Kmer |
| 'GAGTG' | Kmer |
| 'GAGTT' | Kmer |
| 'GATAA' | Kmer |
| 'GATAC' | Kmer |
| 'GATAG' | Kmer |
| 'GATAT' | Kmer |
| 'GATCA' | Kmer |
| 'GATCC' | Kmer |
| 'GATCG' | Kmer |
| 'GATCT' | Kmer |
| 'GATGA' | Kmer |
| 'GATGC' | Kmer |
| 'GATGG' | Kmer |
| 'GATGT' | Kmer |
| 'GATTA' | Kmer |
| 'GATTC' | Kmer |
| 'GATTG' | Kmer |
| 'GATTT' | Kmer |
| 'GCAAA' | Kmer |
| 'GCAAC' | Kmer |
| 'GCAAG' | Kmer |
| 'GCAAT' | Kmer |
| 'GCACA' | Kmer |
| 'GCACC' | Kmer |
| 'GCACG' | Kmer |
| 'GCACT' | Kmer |
| 'GCAGA' | Kmer |
| 'GCAGC' | Kmer |
| 'GCAGG' | Kmer |
| 'GCAGT' | Kmer |
| 'GCATA' | Kmer |
| 'GCATC' | Kmer |
| 'GCATG' | Kmer |
| 'GCATT' | Kmer |
| 'GCCAA' | Kmer |
| 'GCCAC' | Kmer |
| 'GCCAG' | Kmer |
| 'GCCAT' | Kmer |
| 'GCCCA' | Kmer |
| 'GCCCC' | Kmer |
| 'GCCCG' | Kmer |
| 'GCCCT' | Kmer |
| 'GCCGA' | Kmer |
| 'GCCGC' | Kmer |
| 'GCCGG' | Kmer |
| 'GCCGT' | Kmer |
| 'GCCTA' | Kmer |
| 'GCCTC' | Kmer |
| 'GCCTG' | Kmer |
| 'GCCTT' | Kmer |
| 'GCGAA' | Kmer |
| 'GCGAC' | Kmer |
| 'GCGAG' | Kmer |
| 'GCGAT' | Kmer |
| 'GCGCA' | Kmer |
| 'GCGCC' | Kmer |
| 'GCGCG' | Kmer |
| 'GCGCT' | Kmer |
| 'GCGGA' | Kmer |
| 'GCGGC' | Kmer |
| 'GCGGG' | Kmer |
| 'GCGGT' | Kmer |
| 'GCGTA' | Kmer |
| 'GCGTC' | Kmer |
| 'GCGTG' | Kmer |
| 'GCGTT' | Kmer |
| 'GCTAA' | Kmer |
| 'GCTAC' | Kmer |
| 'GCTAG' | Kmer |
| 'GCTAT' | Kmer |
| 'GCTCA' | Kmer |
| 'GCTCC' | Kmer |
| 'GCTCG' | Kmer |
| 'GCTCT' | Kmer |
| 'GCTGA' | Kmer |
| 'GCTGC' | Kmer |
| 'GCTGG' | Kmer |
| 'GCTGT' | Kmer |
| 'GCTTA' | Kmer |
| 'GCTTC' | Kmer |
| 'GCTTG' | Kmer |
| 'GCTTT' | Kmer |
| 'GGAAA' | Kmer |
| 'GGAAC' | Kmer |
| 'GGAAG' | Kmer |
| 'GGAAT' | Kmer |
| 'GGACA' | Kmer |
| 'GGACC' | Kmer |
| 'GGACG' | Kmer |
| 'GGACT' | Kmer |
| 'GGAGA' | Kmer |
| 'GGAGC' | Kmer |
| 'GGAGG' | Kmer |
| 'GGAGT' | Kmer |
| 'GGATA' | Kmer |
| 'GGATC' | Kmer |
| 'GGATG' | Kmer |
| 'GGATT' | Kmer |
| 'GGCAA' | Kmer |
| 'GGCAC' | Kmer |
| 'GGCAG' | Kmer |
| 'GGCAT' | Kmer |
| 'GGCCA' | Kmer |
| 'GGCCC' | Kmer |
| 'GGCCG' | Kmer |
| 'GGCCT' | Kmer |
| 'GGCGA' | Kmer |
| 'GGCGC' | Kmer |
| 'GGCGG' | Kmer |
| 'GGCGT' | Kmer |
| 'GGCTA' | Kmer |
| 'GGCTC' | Kmer |
| 'GGCTG' | Kmer |
| 'GGCTT' | Kmer |
| 'GGGAA' | Kmer |
| 'GGGAC' | Kmer |
| 'GGGAG' | Kmer |
| 'GGGAT' | Kmer |
| 'GGGCA' | Kmer |
| 'GGGCC' | Kmer |
| 'GGGCG' | Kmer |
| 'GGGCT' | Kmer |
| 'GGGGA' | Kmer |
| 'GGGGC' | Kmer |
| 'GGGGG' | Kmer |
| 'GGGGT' | Kmer |
| 'GGGTA' | Kmer |
| 'GGGTC' | Kmer |
| 'GGGTG' | Kmer |
| 'GGGTT' | Kmer |
| 'GGTAA' | Kmer |
| 'GGTAC' | Kmer |
| 'GGTAG' | Kmer |
| 'GGTAT' | Kmer |
| 'GGTCA' | Kmer |
| 'GGTCC' | Kmer |
| 'GGTCG' | Kmer |
| 'GGTCT' | Kmer |
| 'GGTGA' | Kmer |
| 'GGTGC' | Kmer |
| 'GGTGG' | Kmer |
| 'GGTGT' | Kmer |
| 'GGTTA' | Kmer |
| 'GGTTC' | Kmer |
| 'GGTTG' | Kmer |
| 'GGTTT' | Kmer |
| 'GTAAA' | Kmer |
| 'GTAAC' | Kmer |
| 'GTAAG' | Kmer |
| 'GTAAT' | Kmer |
| 'GTACA' | Kmer |
| 'GTACC' | Kmer |
| 'GTACG' | Kmer |
| 'GTACT' | Kmer |
| 'GTAGA' | Kmer |
| 'GTAGC' | Kmer |
| 'GTAGG' | Kmer |
| 'GTAGT' | Kmer |
| 'GTATA' | Kmer |
| 'GTATC' | Kmer |
| 'GTATG' | Kmer |
| 'GTATT' | Kmer |
| 'GTCAA' | Kmer |
| 'GTCAC' | Kmer |
| 'GTCAG' | Kmer |
| 'GTCAT' | Kmer |
| 'GTCCA' | Kmer |
| 'GTCCC' | Kmer |
| 'GTCCG' | Kmer |
| 'GTCCT' | Kmer |
| 'GTCGA' | Kmer |
| 'GTCGC' | Kmer |
| 'GTCGG' | Kmer |
| 'GTCGT' | Kmer |
| 'GTCTA' | Kmer |
| 'GTCTC' | Kmer |
| 'GTCTG' | Kmer |
| 'GTCTT' | Kmer |
| 'GTGAA' | Kmer |
| 'GTGAC' | Kmer |
| 'GTGAG' | Kmer |
| 'GTGAT' | Kmer |
| 'GTGCA' | Kmer |
| 'GTGCC' | Kmer |
| 'GTGCG' | Kmer |
| 'GTGCT' | Kmer |
| 'GTGGA' | Kmer |
| 'GTGGC' | Kmer |
| 'GTGGG' | Kmer |
| 'GTGGT' | Kmer |
| 'GTGTA' | Kmer |
| 'GTGTC' | Kmer |
| 'GTGTG' | Kmer |
| 'GTGTT' | Kmer |
| 'GTTAA' | Kmer |
| 'GTTAC' | Kmer |
| 'GTTAG' | Kmer |
| 'GTTAT' | Kmer |
| 'GTTCA' | Kmer |
| 'GTTCC' | Kmer |
| 'GTTCG' | Kmer |
| 'GTTCT' | Kmer |
| 'GTTGA' | Kmer |
| 'GTTGC' | Kmer |
| 'GTTGG' | Kmer |
| 'GTTGT' | Kmer |
| 'GTTTA' | Kmer |
| 'GTTTC' | Kmer |
| 'GTTTG' | Kmer |
| 'GTTTT' | Kmer |
| 'TAAAA' | Kmer |
| 'TAAAC' | Kmer |
| 'TAAAG' | Kmer |
| 'TAAAT' | Kmer |
| 'TAACA' | Kmer |
| 'TAACC' | Kmer |
| 'TAACG' | Kmer |
| 'TAACT' | Kmer |
| 'TAAGA' | Kmer |
| 'TAAGC' | Kmer |
| 'TAAGG' | Kmer |
| 'TAAGT' | Kmer |
| 'TAATA' | Kmer |
| 'TAATC' | Kmer |
| 'TAATG' | Kmer |
| 'TAATT' | Kmer |
| 'TACAA' | Kmer |
| 'TACAC' | Kmer |
| 'TACAG' | Kmer |
| 'TACAT' | Kmer |
| 'TACCA' | Kmer |
| 'TACCC' | Kmer |
| 'TACCG' | Kmer |
| 'TACCT' | Kmer |
| 'TACGA' | Kmer |
| 'TACGC' | Kmer |
| 'TACGG' | Kmer |
| 'TACGT' | Kmer |
| 'TACTA' | Kmer |
| 'TACTC' | Kmer |
| 'TACTG' | Kmer |
| 'TACTT' | Kmer |
| 'TAGAA' | Kmer |
| 'TAGAC' | Kmer |
| 'TAGAG' | Kmer |
| 'TAGAT' | Kmer |
| 'TAGCA' | Kmer |
| 'TAGCC' | Kmer |
| 'TAGCG' | Kmer |
| 'TAGCT' | Kmer |
| 'TAGGA' | Kmer |
| 'TAGGC' | Kmer |
| 'TAGGG' | Kmer |
| 'TAGGT' | Kmer |
| 'TAGTA' | Kmer |
| 'TAGTC' | Kmer |
| 'TAGTG' | Kmer |
| 'TAGTT' | Kmer |
| 'TATAA' | Kmer |
| 'TATAC' | Kmer |
| 'TATAG' | Kmer |
| 'TATAT' | Kmer |
| 'TATCA' | Kmer |
| 'TATCC' | Kmer |
| 'TATCG' | Kmer |
| 'TATCT' | Kmer |
| 'TATGA' | Kmer |
| 'TATGC' | Kmer |
| 'TATGG' | Kmer |
| 'TATGT' | Kmer |
| 'TATTA' | Kmer |
| 'TATTC' | Kmer |
| 'TATTG' | Kmer |
| 'TATTT' | Kmer |
| 'TCAAA' | Kmer |
| 'TCAAC' | Kmer |
| 'TCAAG' | Kmer |
| 'TCAAT' | Kmer |
| 'TCACA' | Kmer |
| 'TCACC' | Kmer |
| 'TCACG' | Kmer |
| 'TCACT' | Kmer |
| 'TCAGA' | Kmer |
| 'TCAGC' | Kmer |
| 'TCAGG' | Kmer |
| 'TCAGT' | Kmer |
| 'TCATA' | Kmer |
| 'TCATC' | Kmer |
| 'TCATG' | Kmer |
| 'TCATT' | Kmer |
| 'TCCAA' | Kmer |
| 'TCCAC' | Kmer |
| 'TCCAG' | Kmer |
| 'TCCAT' | Kmer |
| 'TCCCA' | Kmer |
| 'TCCCC' | Kmer |
| 'TCCCG' | Kmer |
| 'TCCCT' | Kmer |
| 'TCCGA' | Kmer |
| 'TCCGC' | Kmer |
| 'TCCGG' | Kmer |
| 'TCCGT' | Kmer |
| 'TCCTA' | Kmer |
| 'TCCTC' | Kmer |
| 'TCCTG' | Kmer |
| 'TCCTT' | Kmer |
| 'TCGAA' | Kmer |
| 'TCGAC' | Kmer |
| 'TCGAG' | Kmer |
| 'TCGAT' | Kmer |
| 'TCGCA' | Kmer |
| 'TCGCC' | Kmer |
| 'TCGCG' | Kmer |
| 'TCGCT' | Kmer |
| 'TCGGA' | Kmer |
| 'TCGGC' | Kmer |
| 'TCGGG' | Kmer |
| 'TCGGT' | Kmer |
| 'TCGTA' | Kmer |
| 'TCGTC' | Kmer |
| 'TCGTG' | Kmer |
| 'TCGTT' | Kmer |
| 'TCTAA' | Kmer |
| 'TCTAC' | Kmer |
| 'TCTAG' | Kmer |
| 'TCTAT' | Kmer |
| 'TCTCA' | Kmer |
| 'TCTCC' | Kmer |
| 'TCTCG' | Kmer |
| 'TCTCT' | Kmer |
| 'TCTGA' | Kmer |
| 'TCTGC' | Kmer |
| 'TCTGG' | Kmer |
| 'TCTGT' | Kmer |
| 'TCTTA' | Kmer |
| 'TCTTC' | Kmer |
| 'TCTTG' | Kmer |
| 'TCTTT' | Kmer |
| 'TGAAA' | Kmer |
| 'TGAAC' | Kmer |
| 'TGAAG' | Kmer |
| 'TGAAT' | Kmer |
| 'TGACA' | Kmer |
| 'TGACC' | Kmer |
| 'TGACG' | Kmer |
| 'TGACT' | Kmer |
| 'TGAGA' | Kmer |
| 'TGAGC' | Kmer |
| 'TGAGG' | Kmer |
| 'TGAGT' | Kmer |
| 'TGATA' | Kmer |
| 'TGATC' | Kmer |
| 'TGATG' | Kmer |
| 'TGATT' | Kmer |
| 'TGCAA' | Kmer |
| 'TGCAC' | Kmer |
| 'TGCAG' | Kmer |
| 'TGCAT' | Kmer |
| 'TGCCA' | Kmer |
| 'TGCCC' | Kmer |
| 'TGCCG' | Kmer |
| 'TGCCT' | Kmer |
| 'TGCGA' | Kmer |
| 'TGCGC' | Kmer |
| 'TGCGG' | Kmer |
| 'TGCGT' | Kmer |
| 'TGCTA' | Kmer |
| 'TGCTC' | Kmer |
| 'TGCTG' | Kmer |
| 'TGCTT' | Kmer |
| 'TGGAA' | Kmer |
| 'TGGAC' | Kmer |
| 'TGGAG' | Kmer |
| 'TGGAT' | Kmer |
| 'TGGCA' | Kmer |
| 'TGGCC' | Kmer |
| 'TGGCG' | Kmer |
| 'TGGCT' | Kmer |
| 'TGGGA' | Kmer |
| 'TGGGC' | Kmer |
| 'TGGGG' | Kmer |
| 'TGGGT' | Kmer |
| 'TGGTA' | Kmer |
| 'TGGTC' | Kmer |
| 'TGGTG' | Kmer |
| 'TGGTT' | Kmer |
| 'TGTAA' | Kmer |
| 'TGTAC' | Kmer |
| 'TGTAG' | Kmer |
| 'TGTAT' | Kmer |
| 'TGTCA' | Kmer |
| 'TGTCC' | Kmer |
| 'TGTCG' | Kmer |
| 'TGTCT' | Kmer |
| 'TGTGA' | Kmer |
| 'TGTGC' | Kmer |
| 'TGTGG' | Kmer |
| 'TGTGT' | Kmer |
| 'TGTTA' | Kmer |
| 'TGTTC' | Kmer |
| 'TGTTG' | Kmer |
| 'TGTTT' | Kmer |
| 'TTAAA' | Kmer |
| 'TTAAC' | Kmer |
| 'TTAAG' | Kmer |
| 'TTAAT' | Kmer |
| 'TTACA' | Kmer |
| 'TTACC' | Kmer |
| 'TTACG' | Kmer |
| 'TTACT' | Kmer |
| 'TTAGA' | Kmer |
| 'TTAGC' | Kmer |
| 'TTAGG' | Kmer |
| 'TTAGT' | Kmer |
| 'TTATA' | Kmer |
| 'TTATC' | Kmer |
| 'TTATG' | Kmer |
| 'TTATT' | Kmer |
| 'TTCAA' | Kmer |
| 'TTCAC' | Kmer |
| 'TTCAG' | Kmer |
| 'TTCAT' | Kmer |
| 'TTCCA' | Kmer |
| 'TTCCC' | Kmer |
| 'TTCCG' | Kmer |
| 'TTCCT' | Kmer |
| 'TTCGA' | Kmer |
| 'TTCGC' | Kmer |
| 'TTCGG' | Kmer |
| 'TTCGT' | Kmer |
| 'TTCTA' | Kmer |
| 'TTCTC' | Kmer |
| 'TTCTG' | Kmer |
| 'TTCTT' | Kmer |
| 'TTGAA' | Kmer |
| 'TTGAC' | Kmer |
| 'TTGAG' | Kmer |
| 'TTGAT' | Kmer |
| 'TTGCA' | Kmer |
| 'TTGCC' | Kmer |
| 'TTGCG' | Kmer |
| 'TTGCT' | Kmer |
| 'TTGGA' | Kmer |
| 'TTGGC' | Kmer |
| 'TTGGG' | Kmer |
| 'TTGGT' | Kmer |
| 'TTGTA' | Kmer |
| 'TTGTC' | Kmer |
| 'TTGTG' | Kmer |
| 'TTGTT' | Kmer |
| 'TTTAA' | Kmer |
| 'TTTAC' | Kmer |
| 'TTTAG' | Kmer |
| 'TTTAT' | Kmer |
| 'TTTCA' | Kmer |
| 'TTTCC' | Kmer |
| 'TTTCG' | Kmer |
| 'TTTCT' | Kmer |
| 'TTTGA' | Kmer |
| 'TTTGC' | Kmer |
| 'TTTGG' | Kmer |
| 'TTTGT' | Kmer |
| 'TTTTA' | Kmer |
| 'TTTTC' | Kmer |
| 'TTTTG' | Kmer |
| 'TTTTT' | Kmer |
| 'Pse_AC' | PseKNC |
| 'Pse_CA' | PseKNC |
| 'Pse_TG' | PseKNC |
| 'Pse_lamada_1' | PseKNC |
| 'Pse_lamada_2' | PseKNC |
| 'Pse_lamada_3' | PseKNC |
| 'Pse_lamada_4' | PseKNC |
| 'Pse_lamada_5' | PseKNC |
| 'Pse_lamada_6' | PseKNC |
| 'Pse_lamada_7' | PseKNC |
| 'Pse_lamada_8' | PseKNC |
| 'Pse_lamada_9' | PseKNC |
| 'Pse_lamada_10' | PseKNC |
| 'Pos_1_AAx' | Z_curve_144_bit |
| 'Pos_1_AAy' | Z_curve_144_bit |
| 'Pos_1_AAz' | Z_curve_144_bit |
| 'Pos_2_AAx' | Z_curve_144_bit |
| 'Pos_2_AAy' | Z_curve_144_bit |
| 'Pos_2_AAz' | Z_curve_144_bit |
| 'Pos_3_AAx' | Z_curve_144_bit |
| 'Pos_3_AAy' | Z_curve_144_bit |
| 'Pos_3_AAz' | Z_curve_144_bit |
| 'Pos_1_ACx' | Z_curve_144_bit |
| 'Pos_1_ACy' | Z_curve_144_bit |
| 'Pos_1_ACz' | Z_curve_144_bit |
| 'Pos_2_ACx' | Z_curve_144_bit |
| 'Pos_2_ACy' | Z_curve_144_bit |
| 'Pos_2_ACz' | Z_curve_144_bit |
| 'Pos_3_ACx' | Z_curve_144_bit |
| 'Pos_3_ACy' | Z_curve_144_bit |
| 'Pos_3_ACz' | Z_curve_144_bit |
| 'Pos_1_AGx' | Z_curve_144_bit |
| 'Pos_1_AGy' | Z_curve_144_bit |
| 'Pos_1_AGz' | Z_curve_144_bit |
| 'Pos_2_AGx' | Z_curve_144_bit |
| 'Pos_2_AGy' | Z_curve_144_bit |
| 'Pos_2_AGz' | Z_curve_144_bit |
| 'Pos_3_AGx' | Z_curve_144_bit |
| 'Pos_3_AGy' | Z_curve_144_bit |
| 'Pos_3_AGz' | Z_curve_144_bit |
| 'Pos_1_ATx' | Z_curve_144_bit |
| 'Pos_1_ATy' | Z_curve_144_bit |
| 'Pos_1_ATz' | Z_curve_144_bit |
| 'Pos_2_ATx' | Z_curve_144_bit |
| 'Pos_2_ATy' | Z_curve_144_bit |
| 'Pos_2_ATz' | Z_curve_144_bit |
| 'Pos_3_ATx' | Z_curve_144_bit |
| 'Pos_3_ATy' | Z_curve_144_bit |
| 'Pos_3_ATz' | Z_curve_144_bit |
| 'Pos_1_CAx' | Z_curve_144_bit |
| 'Pos_1_CAy' | Z_curve_144_bit |
| 'Pos_1_CAz' | Z_curve_144_bit |
| 'Pos_2_CAx' | Z_curve_144_bit |
| 'Pos_2_CAy' | Z_curve_144_bit |
| 'Pos_2_CAz' | Z_curve_144_bit |
| 'Pos_3_CAx' | Z_curve_144_bit |
| 'Pos_3_CAy' | Z_curve_144_bit |
| 'Pos_3_CAz' | Z_curve_144_bit |
| 'Pos_1_CCx' | Z_curve_144_bit |
| 'Pos_1_CCy' | Z_curve_144_bit |
| 'Pos_1_CCz' | Z_curve_144_bit |
| 'Pos_2_CCx' | Z_curve_144_bit |
| 'Pos_2_CCy' | Z_curve_144_bit |
| 'Pos_2_CCz' | Z_curve_144_bit |
| 'Pos_3_CCx' | Z_curve_144_bit |
| 'Pos_3_CCy' | Z_curve_144_bit |
| 'Pos_3_CCz' | Z_curve_144_bit |
| 'Pos_1_CGx' | Z_curve_144_bit |
| 'Pos_1_CGy' | Z_curve_144_bit |
| 'Pos_1_CGz' | Z_curve_144_bit |
| 'Pos_2_CGx' | Z_curve_144_bit |
| 'Pos_2_CGy' | Z_curve_144_bit |
| 'Pos_2_CGz' | Z_curve_144_bit |
| 'Pos_3_CGx' | Z_curve_144_bit |
| 'Pos_3_CGy' | Z_curve_144_bit |
| 'Pos_3_CGz' | Z_curve_144_bit |
| 'Pos_1_CTx' | Z_curve_144_bit |
| 'Pos_1_CTy' | Z_curve_144_bit |
| 'Pos_1_CTz' | Z_curve_144_bit |
| 'Pos_2_CTx' | Z_curve_144_bit |
| 'Pos_2_CTy' | Z_curve_144_bit |
| 'Pos_2_CTz' | Z_curve_144_bit |
| 'Pos_3_CTx' | Z_curve_144_bit |
| 'Pos_3_CTy' | Z_curve_144_bit |
| 'Pos_3_CTz' | Z_curve_144_bit |
| 'Pos_1_GAx' | Z_curve_144_bit |
| 'Pos_1_GAy' | Z_curve_144_bit |
| 'Pos_1_GAz' | Z_curve_144_bit |
| 'Pos_2_GAx' | Z_curve_144_bit |
| 'Pos_2_GAy' | Z_curve_144_bit |
| 'Pos_2_GAz' | Z_curve_144_bit |
| 'Pos_3_GAx' | Z_curve_144_bit |
| 'Pos_3_GAy' | Z_curve_144_bit |
| 'Pos_3_GAz' | Z_curve_144_bit |
| 'Pos_1_GCx' | Z_curve_144_bit |
| 'Pos_1_GCy' | Z_curve_144_bit |
| 'Pos_1_GCz' | Z_curve_144_bit |
| 'Pos_2_GCx' | Z_curve_144_bit |
| 'Pos_2_GCy' | Z_curve_144_bit |
| 'Pos_2_GCz' | Z_curve_144_bit |
| 'Pos_3_GCx' | Z_curve_144_bit |
| 'Pos_3_GCy' | Z_curve_144_bit |
| 'Pos_3_GCz' | Z_curve_144_bit |
| 'Pos_1_GGx' | Z_curve_144_bit |
| 'Pos_1_GGy' | Z_curve_144_bit |
| 'Pos_1_GGz' | Z_curve_144_bit |
| 'Pos_2_GGx' | Z_curve_144_bit |
| 'Pos_2_GGy' | Z_curve_144_bit |
| 'Pos_2_GGz' | Z_curve_144_bit |
| 'Pos_3_GGx' | Z_curve_144_bit |
| 'Pos_3_GGy' | Z_curve_144_bit |
| 'Pos_3_GGz' | Z_curve_144_bit |
| 'Pos_1_GTx' | Z_curve_144_bit |
| 'Pos_1_GTy' | Z_curve_144_bit |
| 'Pos_1_GTz' | Z_curve_144_bit |
| 'Pos_2_GTx' | Z_curve_144_bit |
| 'Pos_2_GTy' | Z_curve_144_bit |
| 'Pos_2_GTz' | Z_curve_144_bit |
| 'Pos_3_GTx' | Z_curve_144_bit |
| 'Pos_3_GTy' | Z_curve_144_bit |
| 'Pos_3_GTz' | Z_curve_144_bit |
| 'Pos_1_TAx' | Z_curve_144_bit |
| 'Pos_1_TAy' | Z_curve_144_bit |
| 'Pos_1_TAz' | Z_curve_144_bit |
| 'Pos_2_TAx' | Z_curve_144_bit |
| 'Pos_2_TAy' | Z_curve_144_bit |
| 'Pos_2_TAz' | Z_curve_144_bit |
| 'Pos_3_TAx' | Z_curve_144_bit |
| 'Pos_3_TAy' | Z_curve_144_bit |
| 'Pos_3_TAz' | Z_curve_144_bit |
| 'Pos_1_TCx' | Z_curve_144_bit |
| 'Pos_1_TCy' | Z_curve_144_bit |
| 'Pos_1_TCz' | Z_curve_144_bit |
| 'Pos_2_TCx' | Z_curve_144_bit |
| 'Pos_2_TCy' | Z_curve_144_bit |
| 'Pos_2_TCz' | Z_curve_144_bit |
| 'Pos_3_TCx' | Z_curve_144_bit |
| 'Pos_3_TCy' | Z_curve_144_bit |
| 'Pos_3_TCz' | Z_curve_144_bit |
| 'Pos_1_TGx' | Z_curve_144_bit |
| 'Pos_1_TGy' | Z_curve_144_bit |
| 'Pos_1_TGz' | Z_curve_144_bit |
| 'Pos_2_TGx' | Z_curve_144_bit |
| 'Pos_2_TGy' | Z_curve_144_bit |
| 'Pos_2_TGz' | Z_curve_144_bit |
| 'Pos_3_TGx' | Z_curve_144_bit |
| 'Pos_3_TGy' | Z_curve_144_bit |
| 'Pos_3_TGz' | Z_curve_144_bit |
| 'Pos_1_TTx' | Z_curve_144_bit |
| 'Pos_1_TTy' | Z_curve_144_bit |
| 'Pos_1_TTz' | Z_curve_144_bit |
| 'Pos_2_TTx' | Z_curve_144_bit |
| 'Pos_2_TTy' | Z_curve_144_bit |
| 'Pos_2_TTz' | Z_curve_144_bit |
| 'Pos_3_TTx' | Z_curve_144_bit |
| 'Pos_3_TTy' | Z_curve_144_bit |
| 'Pos_3_TTz' | Z_curve_144_bit |
| 'AAx' | Z_curve_48_bit |
| 'AAy' | Z_curve_48_bit |
| 'AAz' | Z_curve_48_bit |
| 'ACx' | Z_curve_48_bit |
| 'ACy' | Z_curve_48_bit |
| 'ACz' | Z_curve_48_bit |
| 'AGx' | Z_curve_48_bit |
| 'AGy' | Z_curve_48_bit |
| 'AGz' | Z_curve_48_bit |
| 'ATx' | Z_curve_48_bit |
| 'ATy' | Z_curve_48_bit |
| 'ATz' | Z_curve_48_bit |
| 'CAx' | Z_curve_48_bit |
| 'CAy' | Z_curve_48_bit |
| 'CAz' | Z_curve_48_bit |
| 'CCx' | Z_curve_48_bit |
| 'CCy' | Z_curve_48_bit |
| 'CCz' | Z_curve_48_bit |
| 'CGx' | Z_curve_48_bit |
| 'CGy' | Z_curve_48_bit |
| 'CGz' | Z_curve_48_bit |
| 'CTx' | Z_curve_48_bit |
| 'CTy' | Z_curve_48_bit |
| 'CTz' | Z_curve_48_bit |
| 'GAx' | Z_curve_48_bit |
| 'GAy' | Z_curve_48_bit |
| 'GAz' | Z_curve_48_bit |
| 'GCx' | Z_curve_48_bit |
| 'GCy' | Z_curve_48_bit |
| 'GCz' | Z_curve_48_bit |
| 'GGx' | Z_curve_48_bit |
| 'GGy' | Z_curve_48_bit |
| 'GGz' | Z_curve_48_bit |
| 'GTx' | Z_curve_48_bit |
| 'GTy' | Z_curve_48_bit |
| 'GTz' | Z_curve_48_bit |
| 'TAx' | Z_curve_48_bit |
| 'TAy' | Z_curve_48_bit |
| 'TAz' | Z_curve_48_bit |
| 'TCx' | Z_curve_48_bit |
| 'TCy' | Z_curve_48_bit |
| 'TCz' | Z_curve_48_bit |
| 'TGx' | Z_curve_48_bit |
| 'TGy' | Z_curve_48_bit |
| 'TGz' | Z_curve_48_bit |
| 'TTx' | Z_curve_48_bit |
| 'TTy' | Z_curve_48_bit |
| 'TTz' | Z_curve_48_bit |
