# Supplementary material for: Unified mRNA Subcellular Localization Predictor based on machine learning techniques
Source: BMC Genomics. 2024 Feb 7;25:151. doi: 10.1186/s12864-024-10077-9 (PMC10848524; doi:10.1186/s12864-024-10077-9)
Supplement: Supplementary file 2 — Additional File 2. Results of ablation study using different ML models. [file 12864_2024_10077_MOESM2_ESM.docx]

**Additional File 02:** Results of ablation study using different ML models

| **Kmer (2-5)** | Precision | Recall | Acc | Specificity | F1 Score |
| --- | --- | --- | --- | --- | --- |
| **CatBoost** |  |  |  |  |  |
| Cytoplasm | 0.74 | 0.88 | 0.82 | 0.77 | 0.80 |
| ER | 0.94 | 0.38 | 0.94 | 1.00 | 0.54 |
| Extracellular | 0.84 | 0.20 | 0.95 | 1.00 | 0.32 |
| Mitochondria | 0.96 | 0.85 | 0.99 | 1.00 | 0.90 |
| Nucleus | 0.81 | 0.87 | 0.87 | 0.87 | 0.84 |
|  |  |  |  |  |  |
| **XGBoost** |  |  |  |  |  |
| Cytoplasm | 0.75 | 0.88 | 0.82 | 0.78 | 0.81 |
| ER | 0.91 | 0.40 | 0.94 | 1.00 | 0.56 |
| Extracellular | 0.85 | 0.19 | 0.95 | 1.00 | 0.31 |
| Mitochondria | 0.95 | 0.84 | 0.99 | 1.00 | 0.89 |
| Nucleus | 0.82 | 0.87 | 0.87 | 0.87 | 0.84 |
|  |  |  |  |  |  |
| **Decision Tree** |  |  |  |  |  |
| Cytoplasm | 0.65 | 0.66 | 0.71 | 0.74 | 0.66 |
| ER | 0.32 | 0.35 | 0.87 | 0.92 | 0.33 |
| Extracellular | 0.17 | 0.17 | 0.91 | 0.95 | 0.17 |
| Mitochondria | 0.62 | 0.55 | 0.98 | 0.99 | 0.58 |
| Nucleus | 0.70 | 0.69 | 0.76 | 0.81 | 0.70 |
|  |  |  |  |  |  |
| **GNB** |  |  |  |  |  |
| Cytoplasm | 0.63 | 0.19 | 0.61 | 0.92 | 0.29 |
| ER | 0.19 | 0.48 | 0.75 | 0.78 | 0.27 |
| Extracellular | 0.21 | 0.34 | 0.89 | 0.92 | 0.26 |
| Mitochondria | 0.38 | 0.96 | 0.95 | 0.95 | 0.54 |
| Nucleus | 0.58 | 0.68 | 0.68 | 0.68 | 0.62 |
|  |  |  |  |  |  |
| **MLP** |  |  |  |  |  |
| Cytoplasm | 0.77 | 0.81 | 0.82 | 0.82 | 0.79 |
| ER | 0.65 | 0.57 | 0.93 | 0.97 | 0.61 |
| Extracellular | 0.38 | 0.19 | 0.94 | 0.98 | 0.26 |
| Mitochondria | 0.92 | 0.80 | 0.99 | 1.00 | 0.86 |
| Nucleus | 0.80 | 0.84 | 0.86 | 0.86 | 0.82 |

| **PseKNC** | **Precision** | **Recall** | **Acc** | **Specificity** | **F1 Score** |
| --- | --- | --- | --- | --- | --- |
| **CatBoost** |  |  |  |  |  |
| Cytoplasm | 0.64 | 0.77 | 0.72 | 0.68 | 0.70 |
| ER | 0.72 | 0.13 | 0.91 | 0.99 | 0.22 |
| Extracellular | 0.53 | 0.07 | 0.94 | 1.00 | 0.13 |
| Mitochondria | 0.83 | 0.63 | 0.99 | 1.00 | 0.71 |
| Nucleus | 0.67 | 0.75 | 0.76 | 0.76 | 0.71 |
|  |  |  |  |  |  |
| **XGBoost** |  |  |  |  |  |
| Cytoplasm | 0.64 | 0.75 | 0.71 | 0.68 | 0.69 |
| ER | 0.66 | 0.15 | 0.91 | 0.99 | 0.25 |
| Extracellular | 0.50 | 0.08 | 0.94 | 1.00 | 0.14 |
| Mitochondria | 0.83 | 0.58 | 0.98 | 1.00 | 0.68 |
| Nucleus | 0.66 | 0.74 | 0.75 | 0.75 | 0.70 |
|  |  |  |  |  |  |
| **Decision Tree** |  |  |  |  |  |
| Cytoplasm | 0.61 | 0.60 | 0.67 | 0.71 | 0.61 |
| ER | 0.28 | 0.30 | 0.86 | 0.92 | 0.29 |
| Extracellular | 0.13 | 0.13 | 0.90 | 0.95 | 0.13 |
| Mitochondria | 0.51 | 0.45 | 0.97 | 0.99 | 0.48 |
| Nucleus | 0.62 | 0.63 | 0.70 | 0.75 | 0.62 |
|  |  |  |  |  |  |
| **GNB** |  |  |  |  |  |
| Cytoplasm | 0.54 | 0.58 | 0.61 | 0.63 | 0.56 |
| ER | 0.13 | 0.00 | 0.90 | 1.00 | 0.00 |
| Extracellular | 0.31 | 0.04 | 0.94 | 1.00 | 0.07 |
| Mitochondria | 0.21 | 0.78 | 0.91 | 0.91 | 0.33 |
| Nucleus | 0.51 | 0.55 | 0.62 | 0.66 | 0.53 |
|  |  |  |  |  |  |
| **MLP** |  |  |  |  |  |
| Cytoplasm | 0.59 | 0.72 | 0.67 | 0.63 | 0.65 |
| ER | 0.32 | 0.03 | 0.90 | 0.99 | 0.06 |
| Extracellular | 0.48 | 0.08 | 0.94 | 0.99 | 0.14 |
| Mitochondria | 0.75 | 0.65 | 0.98 | 0.99 | 0.70 |
| Nucleus | 0.62 | 0.70 | 0.72 | 0.73 | 0.66 |

| **ZCurve144** | **Precision** | **Recall** | **Acc** | **Specificity** | **F1 Score** |
| --- | --- | --- | --- | --- | --- |
| **CatBoost** |  |  |  |  |  |
| Cytoplasm | 0.68 | 0.83 | 0.76 | 0.70 | 0.74 |
| ER | 0.87 | 0.21 | 0.92 | 1.00 | 0.34 |
| Extracellular | 0.77 | 0.05 | 0.94 | 1.00 | 0.09 |
| Mitochondria | 0.96 | 0.92 | 1.00 | 1.00 | 0.94 |
| Nucleus | 0.75 | 0.81 | 0.82 | 0.83 | 0.78 |
|  |  |  |  |  |  |
| **XGBoost** |  |  |  |  |  |
| Cytoplasm | 0.68 | 0.83 | 0.76 | 0.71 | 0.75 |
| ER | 0.81 | 0.24 | 0.92 | 0.99 | 0.37 |
| Extracellular | 0.71 | 0.06 | 0.94 | 1.00 | 0.11 |
| Mitochondria | 0.95 | 0.88 | 1.00 | 1.00 | 0.92 |
| Nucleus | 0.76 | 0.81 | 0.83 | 0.83 | 0.78 |
|  |  |  |  |  |  |
| **Decision Tree** |  |  |  |  |  |
| Cytoplasm | 0.61 | 0.60 | 0.67 | 0.71 | 0.61 |
| ER | 0.27 | 0.28 | 0.86 | 0.92 | 0.28 |
| Extracellular | 0.10 | 0.09 | 0.90 | 0.95 | 0.10 |
| Mitochondria | 0.72 | 0.66 | 0.98 | 0.99 | 0.68 |
| Nucleus | 0.64 | 0.65 | 0.72 | 0.76 | 0.65 |
|  |  |  |  |  |  |
| **GNB** |  |  |  |  |  |
| Cytoplasm | 0.57 | 0.25 | 0.60 | 0.86 | 0.35 |
| ER | 0.19 | 0.39 | 0.78 | 0.82 | 0.26 |
| Extracellular | 0.19 | 0.31 | 0.88 | 0.92 | 0.23 |
| Mitochondria | 0.70 | 0.96 | 0.99 | 0.99 | 0.81 |
| Nucleus | 0.55 | 0.68 | 0.66 | 0.65 | 0.61 |
|  |  |  |  |  |  |
| **MLP** |  |  |  |  |  |
| Cytoplasm | 0.69 | 0.72 | 0.74 | 0.76 | 0.70 |
| ER | 0.47 | 0.43 | 0.90 | 0.95 | 0.45 |
| Extracellular | 0.23 | 0.19 | 0.92 | 0.96 | 0.21 |
| Mitochondria | 0.94 | 0.86 | 0.99 | 1.00 | 0.90 |
| Nucleus | 0.75 | 0.75 | 0.80 | 0.84 | 0.75 |

| **ZCurve44** | Precision | Recall | Acc | Specificity | F1 Score |
| --- | --- | --- | --- | --- | --- |
| **CatBoost** |  |  |  |  |  |
| Cytoplasm | 0.72 | 0.84 | 0.79 | 0.75 | 0.78 |
| ER | 0.81 | 0.33 | 0.93 | 0.99 | 0.47 |
| Extracellular | 0.80 | 0.11 | 0.95 | 1.00 | 0.19 |
| Mitochondria | 0.94 | 0.87 | 0.99 | 1.00 | 0.90 |
| Nucleus | 0.77 | 0.84 | 0.84 | 0.84 | 0.81 |
|  |  |  |  |  |  |
| **XGBoost** |  |  |  |  |  |
| Cytoplasm | 0.72 | 0.85 | 0.80 | 0.76 | 0.78 |
| ER | 0.83 | 0.36 | 0.93 | 0.99 | 0.51 |
| Extracellular | 0.64 | 0.11 | 0.95 | 1.00 | 0.19 |
| Mitochondria | 0.94 | 0.80 | 0.99 | 1.00 | 0.86 |
| Nucleus | 0.78 | 0.84 | 0.84 | 0.84 | 0.81 |
|  |  |  |  |  |  |
| **Decision Tree** |  |  |  |  |  |
| Cytoplasm | 0.65 | 0.65 | 0.70 | 0.74 | 0.65 |
| ER | 0.35 | 0.36 | 0.88 | 0.93 | 0.36 |
| Extracellular | 0.14 | 0.13 | 0.90 | 0.95 | 0.13 |
| Mitochondria | 0.69 | 0.63 | 0.98 | 0.99 | 0.66 |
| Nucleus | 0.68 | 0.69 | 0.75 | 0.79 | 0.68 |
|  |  |  |  |  |  |
| **GNB** |  |  |  |  |  |
| Cytoplasm | 0.60 | 0.31 | 0.62 | 0.85 | 0.41 |
| ER | 0.24 | 0.36 | 0.83 | 0.88 | 0.28 |
| Extracellular | 0.22 | 0.24 | 0.91 | 0.95 | 0.23 |
| Mitochondria | 0.43 | 0.93 | 0.96 | 0.96 | 0.59 |
| Nucleus | 0.55 | 0.73 | 0.66 | 0.62 | 0.63 |
|  |  |  |  |  |  |
| **MLP** |  |  |  |  |  |
| Cytoplasm | 0.70 | 0.75 | 0.76 | 0.76 | 0.73 |
| ER | 0.51 | 0.41 | 0.91 | 0.96 | 0.46 |
| Extracellular | 0.32 | 0.20 | 0.93 | 0.97 | 0.25 |
| Mitochondria | 0.92 | 0.83 | 0.99 | 1.00 | 0.88 |
| Nucleus | 0.75 | 0.78 | 0.81 | 0.83 | 0.76 |
