# Supplementary material for: Unified mRNA Subcellular Localization Predictor based on machine learning techniques
Source: BMC Genomics. 2024 Feb 7;25:151. doi: 10.1186/s12864-024-10077-9 (PMC10848524; doi:10.1186/s12864-024-10077-9)

**Additional File 03:** Motifs identified based on mRNA sequence from different
subcellular localizations.

**Cytoplasm - Motifs**


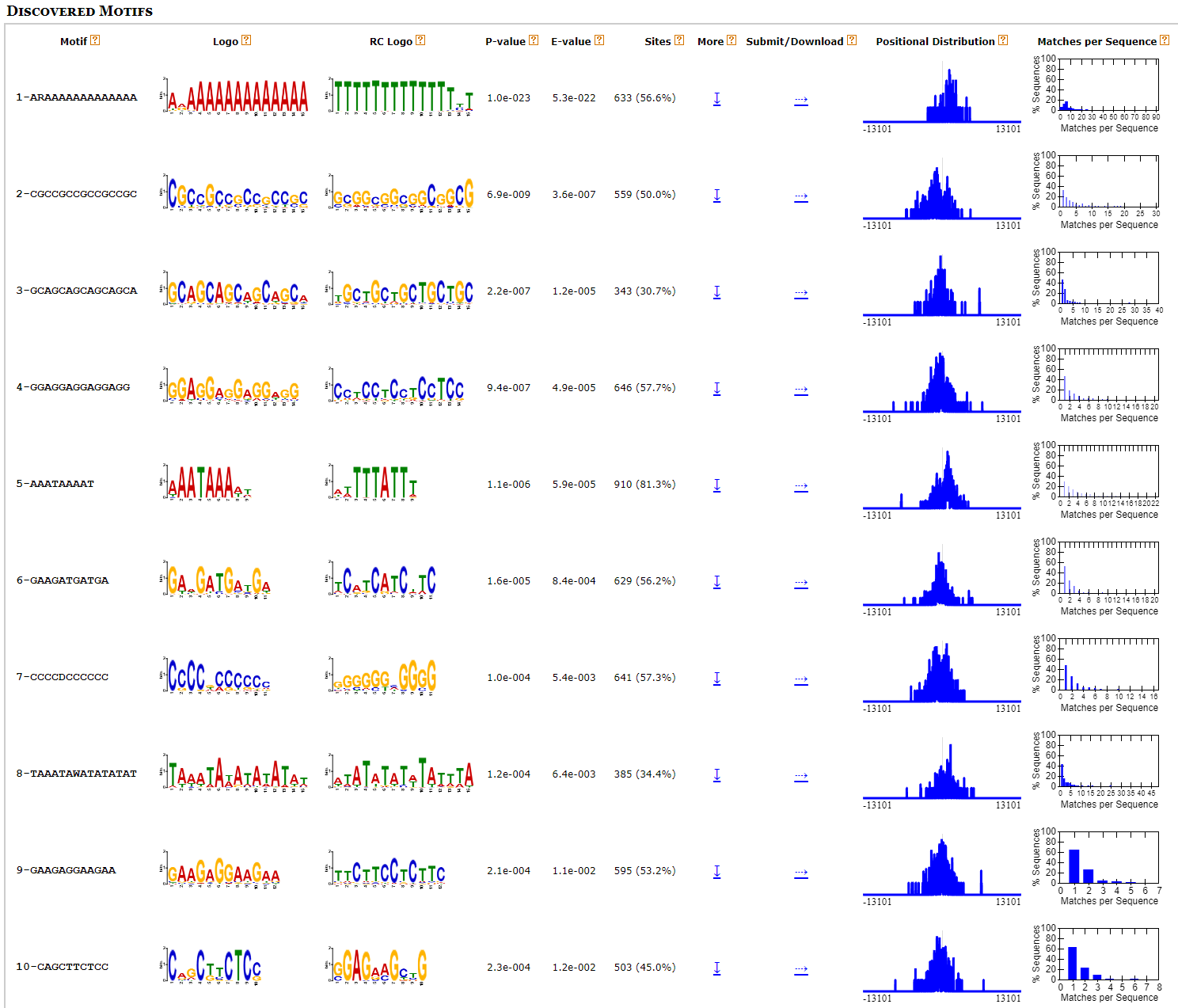


**Nucleus - Motifs**


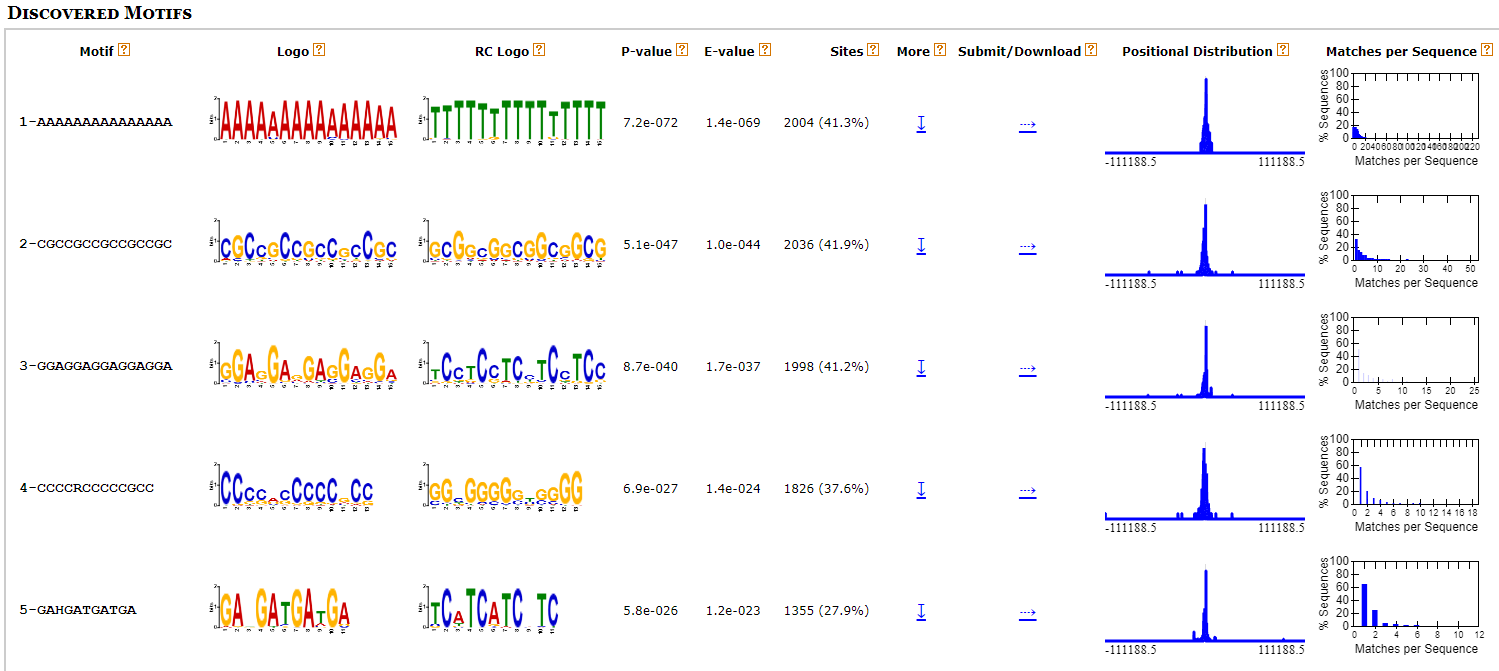


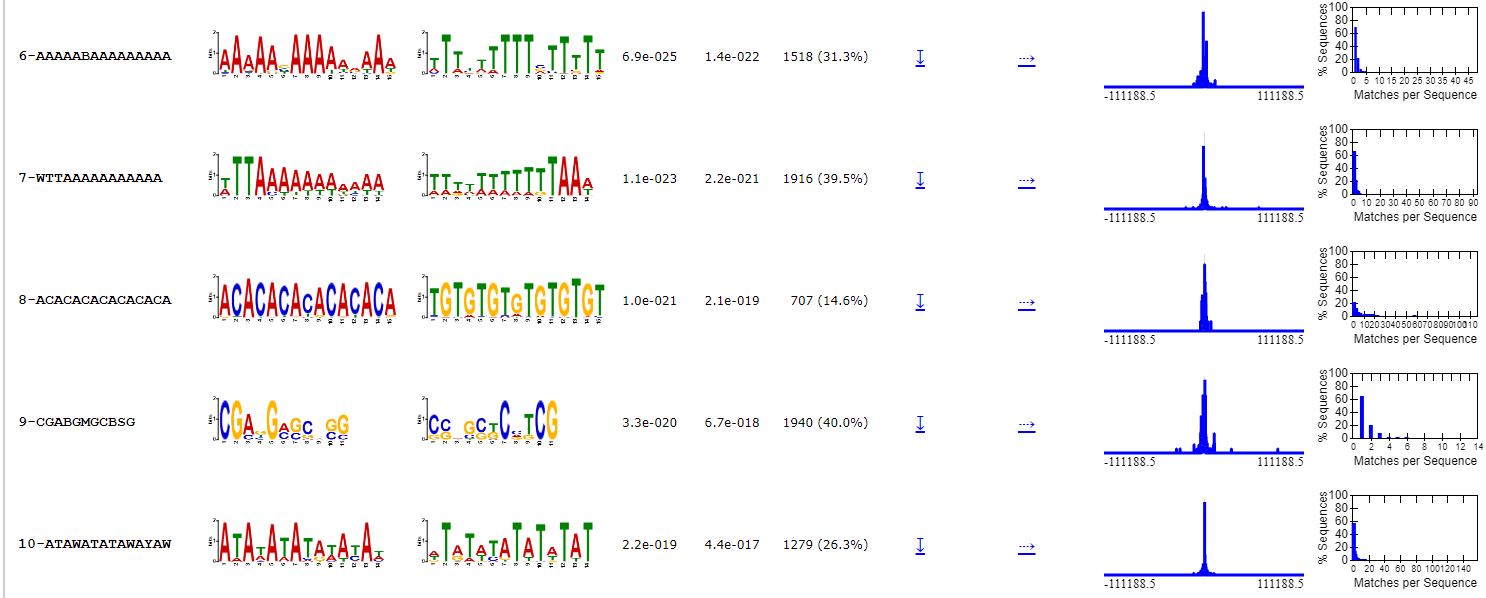


**Mitochondria Motifs**


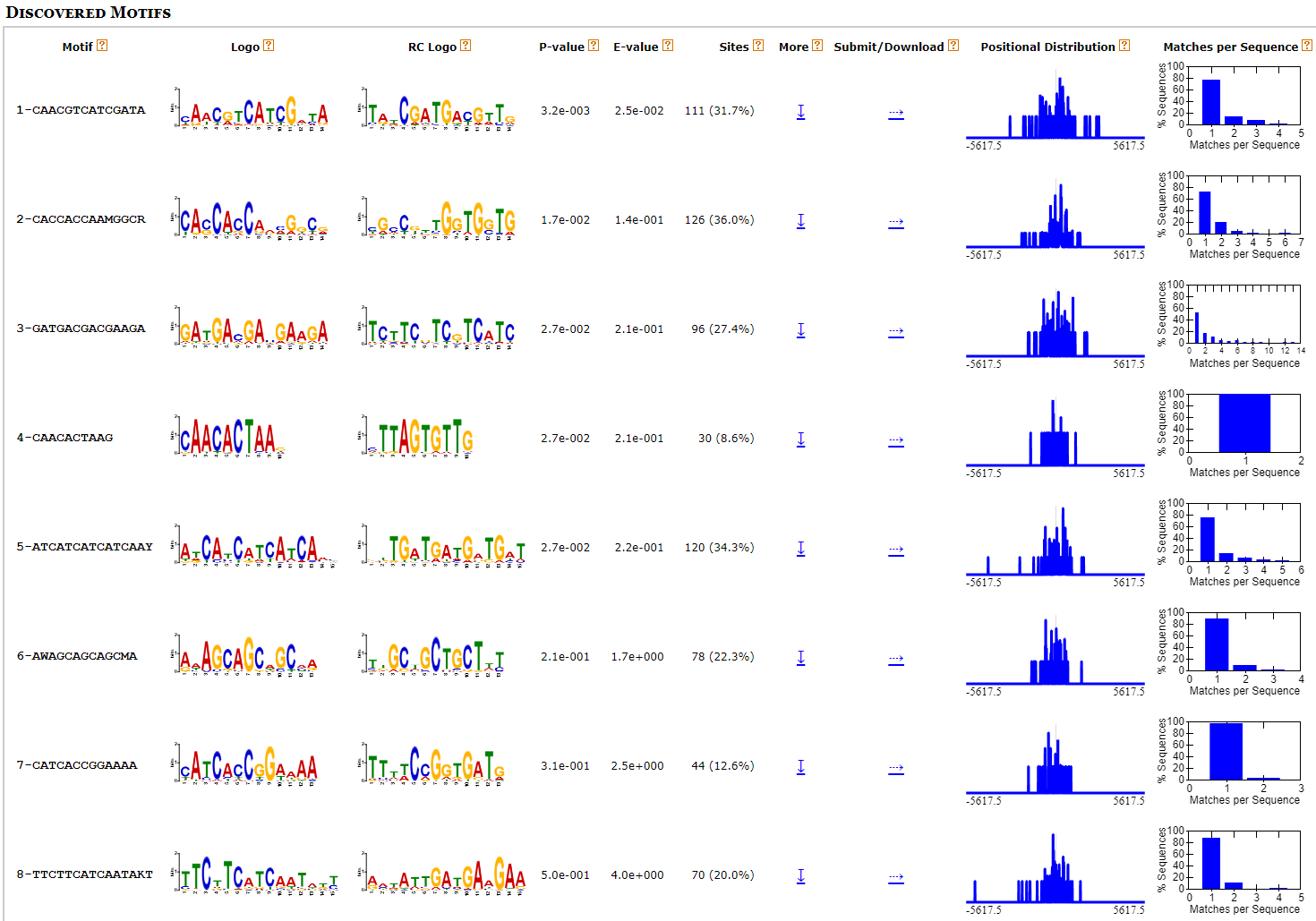


**ExR Motifs**


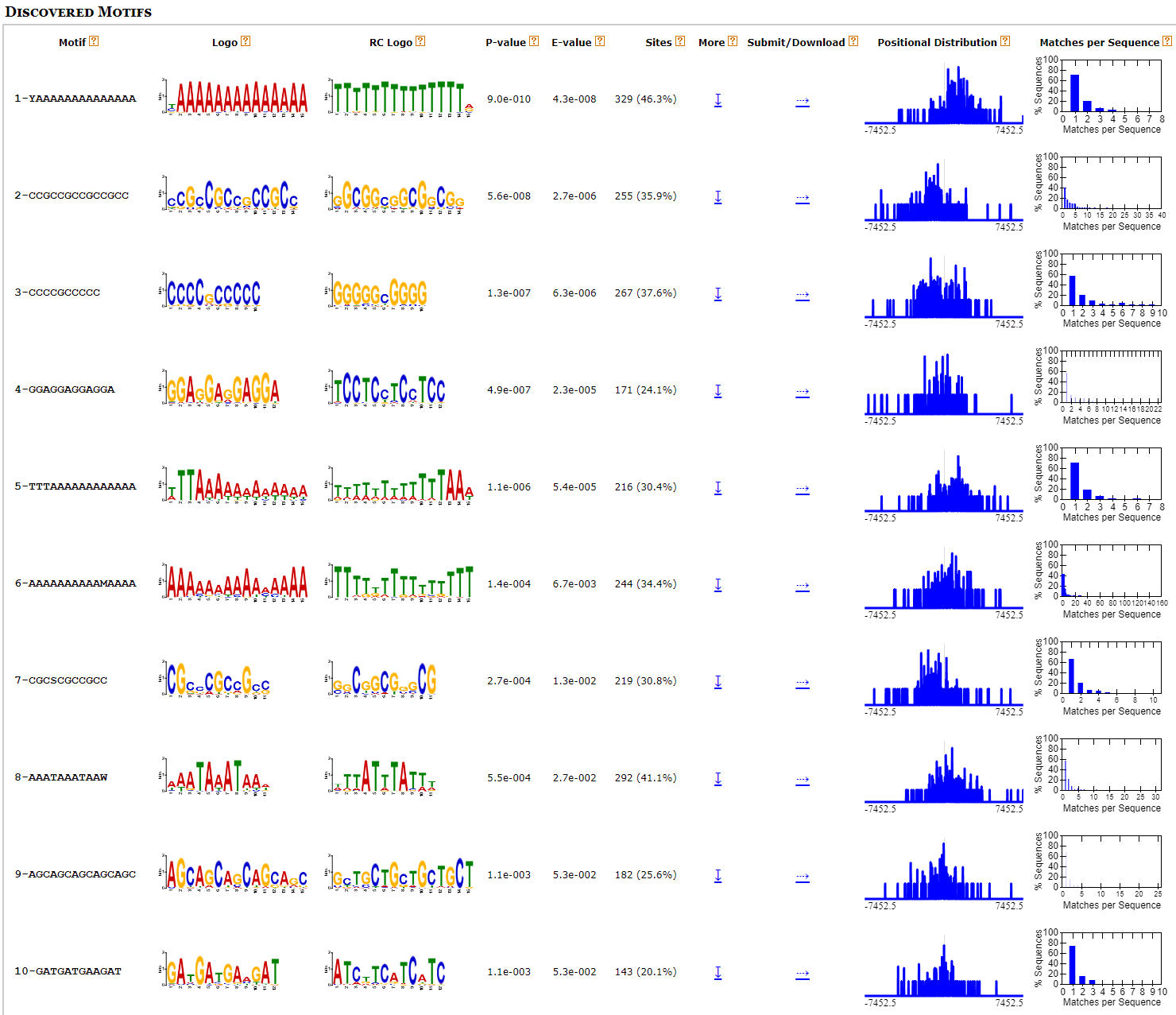


ER


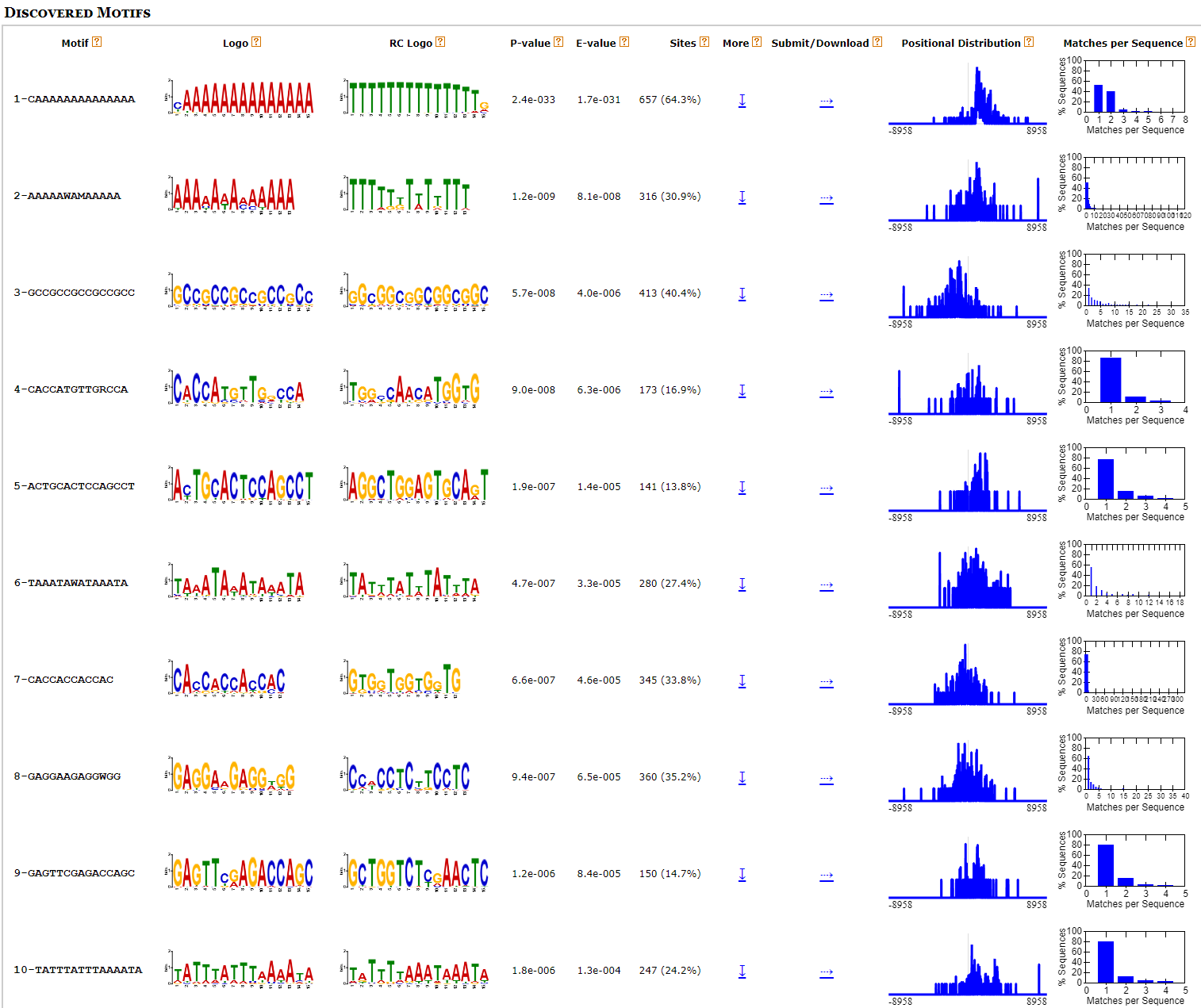

Supplement: Supplementary file 3 — Additional File 3. Motifs identified based on mRNA sequence from different subcellular localizations. [file 12864_2024_10077_MOESM3_ESM.docx]
